# An urgent call to publish COVID-19 trials: a systematic search revealed ZERO studies regarding the incidence of thromboembolic events in SARS-CoV-2 Omicron-infected COVID-19 patients

**DOI:** 10.1186/s13054-025-05395-7

**Published:** 2025-05-01

**Authors:** Amon Faske, Stefanie Reis, Tamara Pscheidl, Lena Saal-Bauernschubert, Patrick Meybohm, Stephanie Weibel

**Affiliations:** Department for Anaesthesiology, Intensive Care, Emergency and Pain Medicine, University Hospital Würzburg, Oberdürrbacher Str. 6, 97080 Würzburg, Germany

**Keywords:** COVID-19, Thrombosis, Omicron, SARS-CoV-2 variants, Systematic review

## Abstract

**Background:**

COVID-19 has been associated with an increased risk of thromboembolic complications, particularly in hospitalized patients. While early research focused on pre-Omicron variants, the thrombotic risk associated with SARS-CoV-2 Omicron infections remains unclear. Given the evolving nature of the pandemic, it is critical to assess whether current anticoagulation recommendations remain appropriate.

**Methods:**

We conducted a systematic review of clinical studies to determine the incidence of thromboembolic events in COVID-19 patients infected with SARS-CoV-2 Omicron variants. The main outcome was thromboembolic events within 28 days of infection, using objective diagnostic criteria. We systematically searched the Cochrane COVID‐19 Study Register, covering multiple databases, for studies published between November 30, 2021, and January 31, 2024. Studies were screened independently by two reviewers, and missing data were requested from study authors.

**Results:**

Our search identified 7843 records, of which 238 underwent full-text screening. Ultimately, no study met our inclusion criteria due to issues such as lack of Omicron-specific data, inadequate reporting of diagnostic methods, and failure to specify the timing of outcome assessment. Despite contacting study authors, no additional eligible data were obtained.

**Conclusion:**

There is currently no high-quality evidence on the incidence of thromboembolic events in Omicron-infected COVID-19 patients. The absence of relevant studies highlights a critical research gap and raises concerns about the applicability of current anticoagulation guidelines. Future studies should stratify outcomes by SARS-CoV-2 variant, ensure transparent reporting, and provide rigorous diagnostic confirmation to guide clinical decision-making.

Reports in the pandemic have shown that COVID-19 is associated with an increased risk of thromboembolic complications, such as pulmonary embolism and deep vein thrombosis [1]. Early studies indicated that this risk is notably higher in patients treated in intensive care units compared to other hospitalized patients, and also suggested that the risk, especially in mild cases and among outpatients, might be lower [1–3]. While early prophylactic anticoagulation might reduce the thromboembolic risk, it remained unclear for a long time whether it can prevent disease progression without causing adverse effects. We therefore conducted a systematic review with meta-analysis of all available randomized controlled trials (RCTs) to determine the safety and efficacy of anticoagulation at any dosage compared with standard low-dose prophylactic anticoagulation or no prophylaxis in COVID-19 patients regardless of disease severity and treatment setting [4]. The published systematic review with search in October 2023 informed the German AWMF-S3 guidelines published in February 2024 addressing anticoagulation in COVID-19 patients, which subsequently recommended anticoagulation strategies based on the severity and setting of COVID-19 infection [5]. However, the evidence has limited transferability to the current situation for two main reasons. First, most studies were based on participants who were predominantly unvaccinated, making the results less applicable to today’s treatment context. Second, the majority of included studies were conducted in 2020–2021, meaning the viral variants involved no longer match those circulating today. Although the incidence of thromboembolic events has been well-studied for earlier SARS-CoV-2 variants, there is limited data on the risk of thrombosis following infection with newer variants, such as Omicron. It remains uncertain whether the Omicron variants continue to pose a significant thrombotic risk, highlighting the need for further investigation. This uncertainty raises the question of whether the recommended anticoagulation strategies are still appropriate given the increased risk for adverse events such as major bleedings.

To close this gap in the evidence, we set out to identify the incidence of thromboembolic events in COVID-19 patients infected with SARS-CoV-2 Omicron variants. We planned a systematic review with meta-analysis and prospectively registered a review protocol on PROSPERO (CRD42024515716, [6]). We considered studies eligible which reported the incidence of thromboembolic events in adult COVID-19 patients with confirmed SARS-CoV-2 infection, independent of disease severity, vaccination status, concomitant thromboprophylaxis or other risk factors. Omicron infection must be explicitly mentioned in the study or was assumed if participants were recruited after December 2021. Studies including non-Omicron periods were also eligible if outcomes for the Omicron subgroup were reported separately. If no subgroups were provided, the study remained eligible if no more than 10% of participants were recruited before December 2021. The two main outcomes were the occurrence of any thromboembolic event including deep vein thrombosis (including catheter-associated), pulmonary embolism, myocardial infarction, arterial ischemia (such as mesenteric or extremity ischemia), acute splanchnic vein thrombosis, or ischemic stroke, and the combined outcome of any thromboembolic event or death. All thromboembolic events must be diagnosed and objectively confirmed using appropriate diagnostic techniques such as angiography, including ultrasound, computer tomography, or magnetic resonance imaging. The time point for outcome measurement was set within 28 days of symptom onset or diagnosis of infection. Questions of incidence are best answered in cohort studies [7]. We considered cohort studies, both prospective and retrospective, as eligible study designs. A minimum sample size of 100 participants was required. We restricted eligible studies to publications in English and German and excluded letters, commentaries, and conference abstracts. A systematic search for eligible studies was conducted in the Cochrane COVID‐19 Study Register (CCSR) [8] comprising several databases, i.e. Cochrane Central Register of Controlled Trials (CENTRAL), MEDLINE (PubMed), Embase, ClinicalTrials.gov, WHO International Clinical Trials Registry Platform (ICTRP), and medRxiv for published studies and registered studies with reported results between 30 November 2021 and 31 January 2024. We did not conduct separate searches of the databases, since these databases are being regularly searched for the production of the CCSR until 31 January 2024. The search term included "thrombotic" or "thrombosis" or "thromboses" or "thromboembolic" or "venous thromboembolism" or "deep vein thrombosis" or "pulmonary embolism" or "coagulopathy" or “ischemic stroke” or “stroke”. Two review authors independently screened the title and abstract of records retrieved by the search. We aimed to obtain the full text of all potentially relevant records, and independently screened them to select studies for inclusion. We resolved disagreements through consensus or by discussion with a third review author. If not all eligibility criteria were fulfilled, we categorised the study as 'awaiting classification' and contacted the study authors for clarification.

We identified 7843 records with our search, removed two duplicates, and deemed 7603 as irrelevant during title and abstract screening (Fig. 1, [9]). The remaining 238 records proceeded to full text screening but one study could not be obtained via the interlibrary loan. After full text screening, none of the 237 screened studies with reported results was fully eligible for inclusion into our systematic review. Initially, 75 reports were classified as ‘awaiting classification’. Contact information was retrieved for 73 studies and author requests resulted in 13 responses. Finally, none of the 13 studies fulfilled our eligibility criteria and were excluded. Altogether, we excluded 175 studies. The three main reasons for exclusion were a wrong publication format (conference abstract, n = 63), a study period before Omicron (n = 52), and reporting of wrong outcomes (n = 22) (Fig. 1). The remaining 62 studies were classified as ‘awaiting classification’ due to missing information on at least one eligibility criteria, i.e. 46 studies did not separately report outcome data for patients infected with the Omicron variant, in four studies the study period was not reported, another four studies did not report the time point of outcome assessment, in three studies the method of outcome confirmation remained unclear, and five studies did not report on time point of outcome assessment and outcome confirmation.

**Fig. 1:**
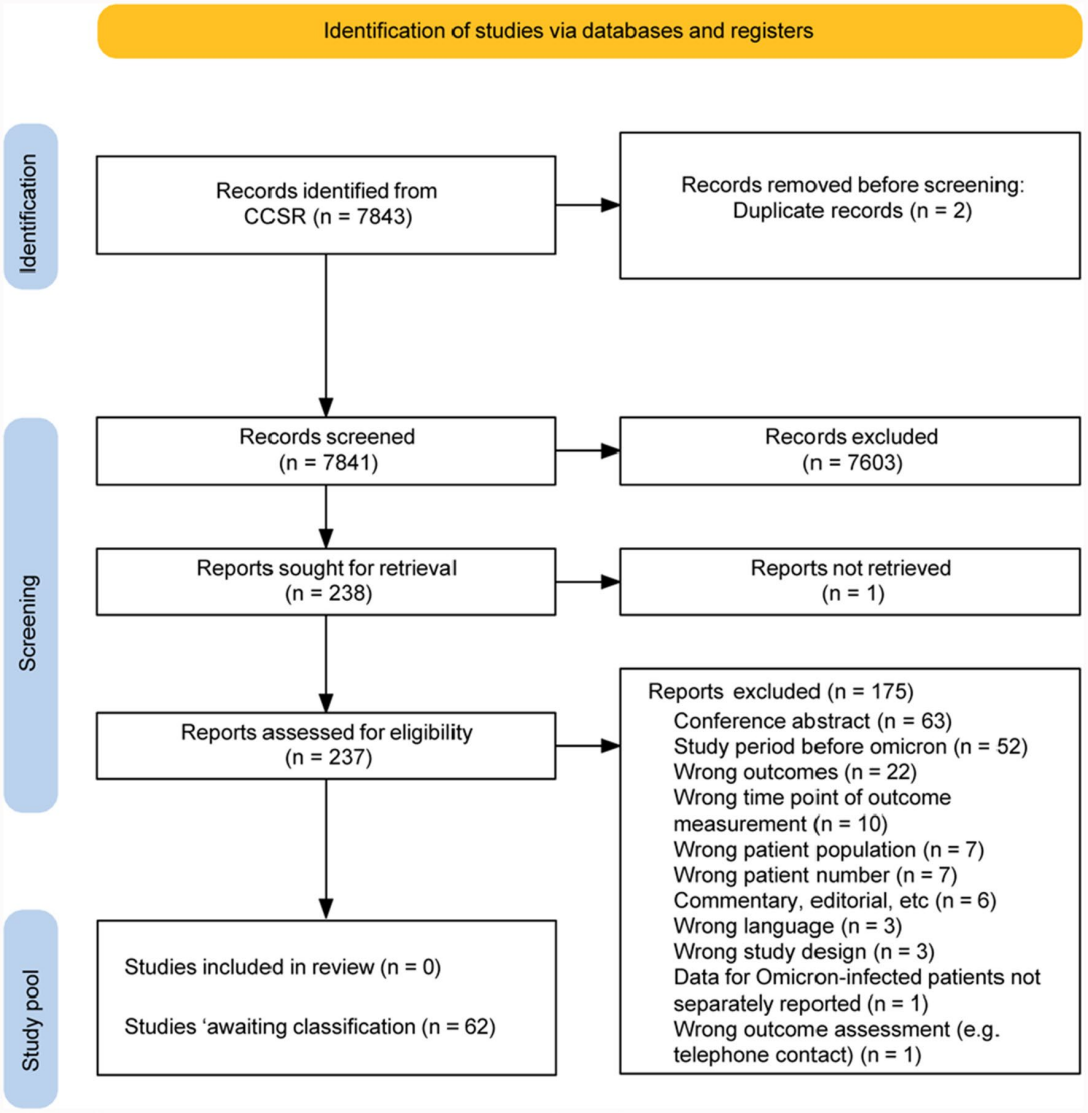
PRISMA flow diagram

We were unable to find an answer to our question about the incidence of thromboembolic events within 28 days of infection in COVID-19 patients in the Omicron era. We were surprised that we were not able to find at least one study that met the eligibility criteria of our systematic review. In contrast to the pre-Omicron era, where a large number of studies on thromboembolic events in COVID-19 patients have been published [10], evidence on the incidence of thromboembolism are lacking in the Omicron era.

There are several possible explanations for our findings. First, it is possible that, at the time of our search, relevant studies investigating Omicron patients had not yet been published. This is supported by the large number of identified conference abstracts. Although these abstracts hint at ongoing research in this area, it must be emphasized they were not evaluated further for inclusion due to our strict methodology. This presents a potential limitation, as some relevant studies may not yet have been fully disseminated or peer-reviewed. Second, it is certainly decisive that many relevant studies do not report separate outcome data for the population of Omicron patients. Despite our efforts to contact study authors and request stratified data, we were unable to obtain data on this population. Consequently, studies that combined data from different variants without distinguishing Omicron patients were excluded. Another issue is our strict inclusion criteria, such as requiring objective diagnostic confirmation of the outcome assessment. This was intended to enhance the rigor and reliability of our analysis, however many studies failed to meet these standards due to insufficient reporting of key details such as the study period, timing of the outcome measurement, or outcome diagnostics were not described. This lack of transparency in reporting hindered our ability to reliably include such studies, potentially leading to the exclusion of relevant data. Another limitation could be the restriction of our search to the CCSR database, which generally covers all relevant databases, but no database-specific searches were conducted. Therefore, it is possible that the search in the CCSR database is less sensitive than searches in the individual databases.

Our study highlights the need for further cohort studies investigating the incidence of thromboembolic events in COVID-19 patients infected with the Omicron variant. We are sending an appeal to all investigators to publish their studies and report the data for Omicron-infected patients separately and provide further details about the outcome measurement. As long as the baseline incidence of thromboembolic events in COVID-19 patients with Omicron infection is unknown, the use of thromboprophylaxis or anticoagulation in hospitalized patients must be carefully weighed against the risk of bleeding.

## Data Availability

No datasets were generated or analysed during the current study. All supporting data are available upon request from the authors.
